# Spontaneous Chest Wall Hematoma in a Hemodialysis Patient: A Case Report

**DOI:** 10.3390/jcm14020396

**Published:** 2025-01-10

**Authors:** Seung-Hyun Kim, Ho-Jun Lee, Dong-Wan Kim, Jun-Ho Choi, Jae-Ha Hwang, Kwang-Seog Kim

**Affiliations:** Department of Plastic and Reconstructive Surgery, Chonnam National University Hospital, Chonnam National University Medical School, 42 Jebong-ro, Dong-gu, Gwangju 61469, Republic of Korea; hi_1004@naver.com (S.-H.K.); lghk0419@naver.com (H.-J.L.); waaan37@gmail.com (D.-W.K.); cjh_0502@hanmail.net (J.-H.C.); pskim@chonnam.ac.kr (K.-S.K.)

**Keywords:** chest wall hematoma, hemodialysis, uremic coagulopathy, surgical evacuation

## Abstract

**Background/Objectives**: Spontaneous chest wall hematomas are rare but potentially life-threatening complications, particularly in patients with multiple comorbidities such as those undergoing hemodialysis. This case report aims to highlight the significance of early diagnosis and appropriate management in preventing complications associated with this condition. **Methods**: We report the case of a 79-year-old man with end-stage renal disease on hemodialysis, presenting with a large spontaneous hematoma (18.7 × 13.1 × 9.6 cm) in the right upper chest wall. Initial interventions, including diagnostic imaging and transarterial angiography, did not detect active bleeding. Surgical exploration revealed bleeding from a branch of the lateral thoracic artery, which was managed through vessel ligation and hematoma drainage. **Results**: The hematoma was successfully managed through surgical intervention, with no postoperative complications such as infection, recurrent bleeding, or wound issues. The patient’s recovery was uneventful, and he was discharged in stable condition. This case underscores the role of microvascular fragility and uremic coagulopathy in the development of spontaneous bleeding in hemodialysis patients. **Conclusions**: This case emphasizes the importance of timely recognition and individualized management of spontaneous soft tissue bleeding in hemodialysis patients. Surgical intervention is necessary for large hematomas or cases of hemodynamic instability to ensure hemostasis and mitigate complications.

## 1. Introduction

Chest wall hematomas are most commonly caused by trauma. However, spontaneous chest wall hematomas, though rare, represent a clinically significant complication. They are frequently seen in patients undergoing anticoagulation therapy or those with comorbidities that increase their bleeding risk, and recent case reports have identified additional factors such as platelet dysfunction in uremic patients and anticoagulation during hemodialysis [1,2,3,4]. These hematomas can develop in the absence of any traumatic event, which complicates both diagnosis and management. Older adults, especially those with underlying conditions such as renal impairment or hypertension, are at an increased risk due to age-related vascular fragility, a higher burden of comorbidities, and potential medication interactions [5].

Fior et al. noted that the most common anatomical locations for these hematomas are the rectus sheath (53%), lower limbs (21%), and iliopsoas muscles (14%) [6]. Hematomas in the chest wall are particularly rare, which makes clinical cases involving this location especially significant [6]. Research has indicated that the use of anticoagulants, especially in older patients with diminished renal function, necessitates careful dosing and vigilant monitoring to avoid excessive bleeding. Furthermore, the literature points to microvascular arteriolar damage as a potential underlying pathophysiological mechanism in such cases, which may be aggravated by high blood pressure and decreased vascular elasticity in older adults [7,8,9].

This report presents the case of a 79-year-old man with multiple comorbidities, including end-stage renal disease (ESRD) and ongoing hemodialysis, who presented with a large spontaneous hematoma in the right upper chest wall. In this case, attempts at transarterial diagnosis and embolization proved unsuccessful, requiring surgical intervention to manage the hematoma and avert further complications.

## 2. Case Presentation

A 79-year-old male patient was admitted to our institution’s nephrology department and subsequently referred to the plastic and reconstructive surgery team for the surgical management of a progressively enlarging, painful mass-like lesion on the right side of his chest, first observed 3 days earlier. The patient had a history of several chronic conditions, including hypertension, diabetes mellitus, hypothyroidism, and adrenal insufficiency. His treatment regimen included azilsartan, nebivolol, nifedipine, doxazosin, teneligliptin, gliclazide, levothyroxine, and hydrocortisone.

Initially, the patient had been hospitalized for care of his general condition following a primary closure for acute panperitonitis and a perihepatic abscess, which had developed secondary to a duodenal perforation identified during an evaluation for abdominal pain. This evaluation occurred while the patient was undergoing hemodialysis for ESRD through a permanent central venous catheter. During hospitalization, a retrogastric fluid collection was identified, prompting an endoscopic ultrasound-guided gastrostomy and the placement of a lumen-apposing metal stent (LAMS). One month later, the LAMS was removed, and endoscopic hemoclipping was performed to address bleeding at the insertion site. Due to new-onset anemia, 4 units of packed red blood cells (PRCs) were transfused. Apixaban was prescribed for paroxysmal atrial fibrillation detected early during hospitalization. However, it was discontinued when gastrointestinal bleeding was identified. After sinus conversion was observed, apixaban was not reintroduced, and no other anticoagulants were administered. His vital signs remained generally stable, although he experienced occasional systolic blood pressure spikes ranging from 170 to 200 mmHg. One month prior to the development of chest wall hematoma, he had been treated for hospital-acquired pneumonia using piperacillin, tazobactam, and metronidazole.

Physical examination revealed tenderness and fluctuation in the lesion, with no signs of bruising. Laboratory tests indicated a hemoglobin level of 8.3 g/dL, an elevated C-reactive protein (CRP) level of 8.04 mg/dL, and a procalcitonin level of 7.05 ng/mL. The coagulation profile, including platelet count, prothrombin time (international normalized ratio [INR]), and activated partial-thromboplastin time, was within the normal range (Table 1).

**Figure 1:**
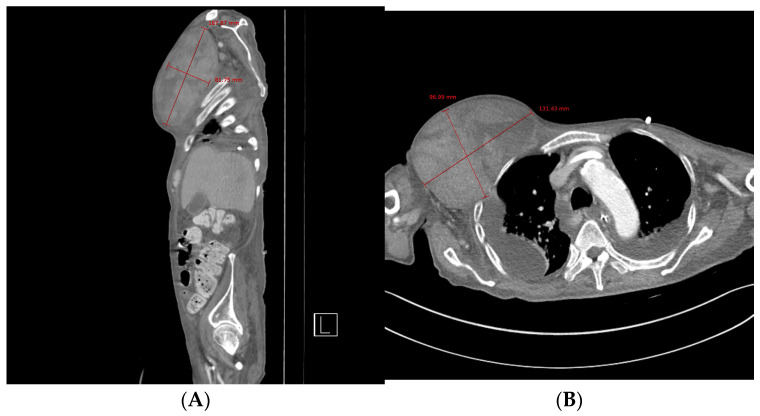
Chest computed tomography. (**A**) Sagittal view showing the formation of a hematoma measuring approximately 19 × 8 cm. (**B**) Axial view showing a newly formed 13 × 10 cm hematoma in the right upper anterior chest wall without evidence of active bleeding or pseudoaneurysm.

**Figure 2:**
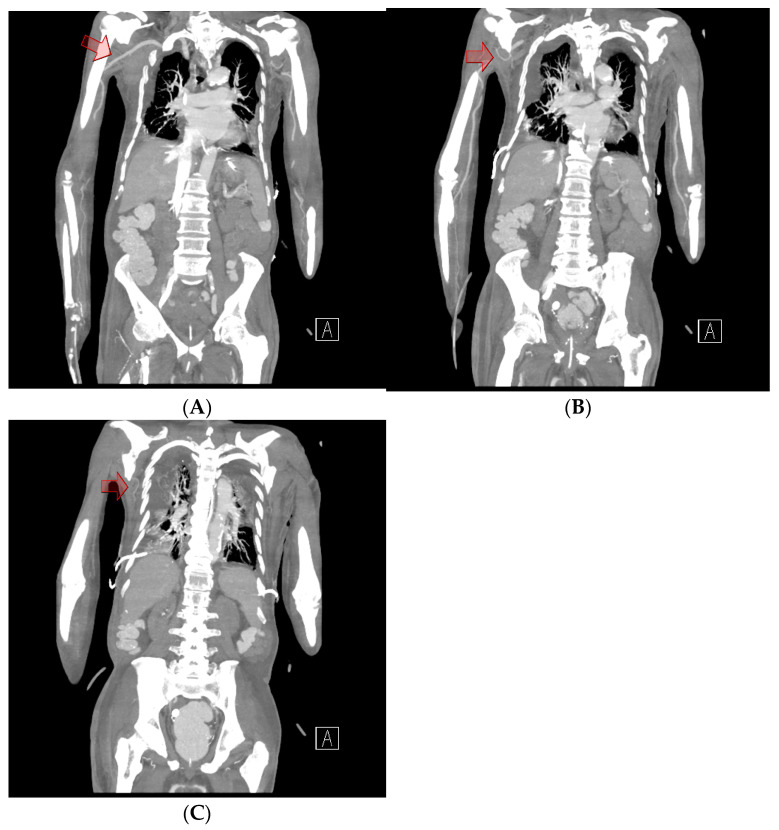
Chest computed tomography angiography. (**A**) A coronal view shows intact vessel patency in the subclavian artery and axillary artery (red arrow), which could have contributed to the hematoma in the chest wall. (**B**) No extravasation or abnormalities were observed in the thoracoacromial artery (red arrow). (**C**) No abnormalities were observed in the main pedicle of the lateral thoracic artery (red arrow).

Chest computed tomography (CT) angiography revealed a newly formed 18.7 × 13.1 × 9.6 cm hematoma in the right upper anterior chest wall, with no signs of active bleeding or evidence of a pseudoaneurysm or malignant tumor (Figure 1 and Figure 2). On the fourth day post-onset, transarterial angiography was conducted via the right common femoral artery. Angiograms of the right subclavian artery, right internal mammary artery, and thoracic aorta showed no active bleeding, precluding the possibility of embolization. To manage the resulting anemia, 2 units of PRCs were transfused.

**Figure 3:**
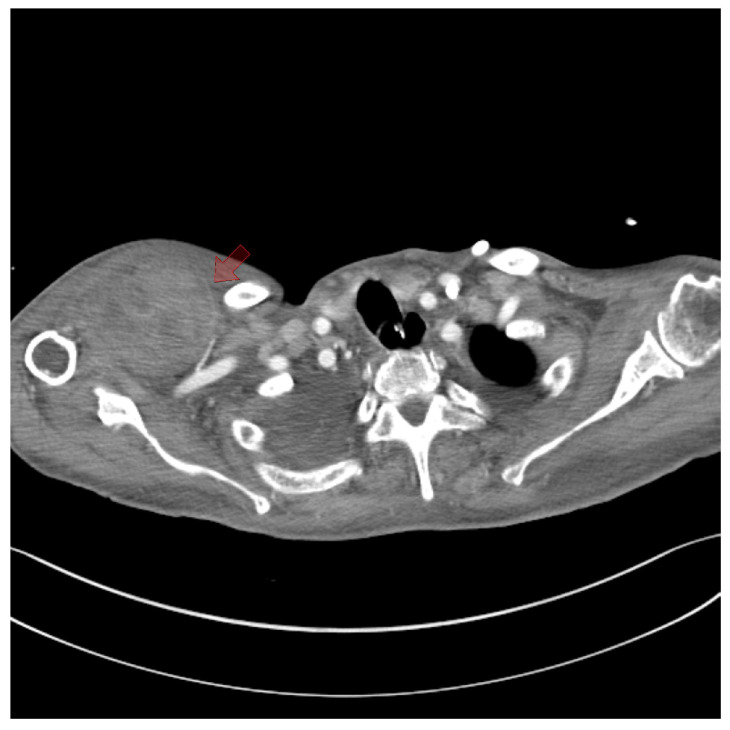
Axial section of the thorax at the axillary level. Computed tomography angiography revealed a branch of the lateral thoracic artery located near the subpectoral hematoma (red arrow).

**Figure 4:**
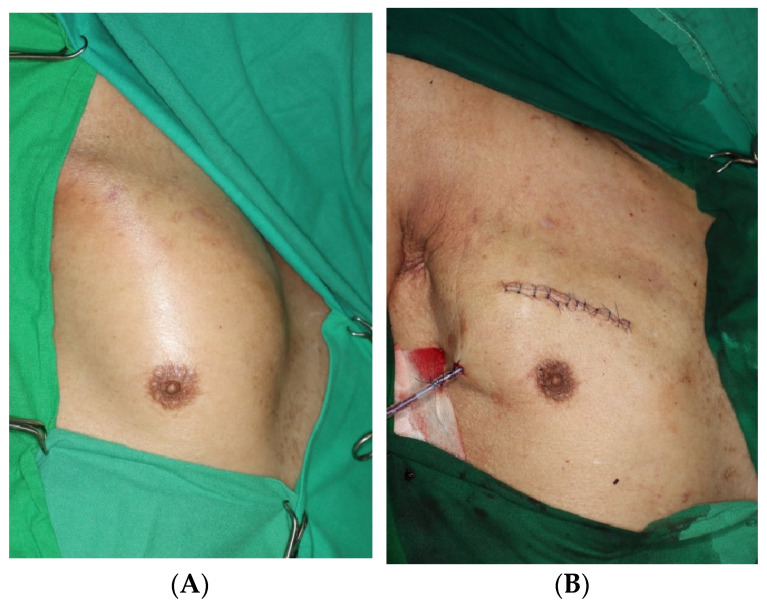
Intraoperative photographs. (**A**) Preoperative photograph. The patient presented with a painful mass-like lesion on the right side of the chest, without any bruising. (**B**) Postoperative photograph. Hematoma removal and vessel ligation were performed, followed by irrigation, placement of a closed suction drain, and primary closure.

On the fifth day, surgical exploration revealed a large hematoma beneath the pectoralis major muscle. The hematoma had solidified and showed no signs of infection; culture results were negative. Bleeding was identified from a branch of the lateral thoracic artery within the deep portion of the pectoralis major muscle, as observed on CT angiography (Figure 3). The procedure involved removing the hematoma and ligating the vessel, followed by irrigation, placement of a closed suction drain, and primary closure. The surgery was completed with a mild compressive dressing (Figure 4).

Postoperatively, there were no signs of bleeding, such as recurrent swelling or a rapid drop in hemoglobin levels. The patient did not experience any complications, including infection, inflammation, skin thinning, wound dehiscence, or seroma formation. All stitches were removed by the second postoperative week, and he was discharged in stable condition by the third week.

## 3. Discussion

As is widely known, ultrasonography and contrast-enhanced CT are recognized as definitive diagnostic tools in evaluating hematomas. The sensitivity of CT angiography, ultrasonography, and arteriography for this purpose is 95.2%, around 90%, and 80%, respectively [10,11,12,13]. Therefore, CT and ultrasonography are most commonly considered as initial diagnostic tools, and the choice between these possibilities can be made based on their advantages and limitations. Specifically, contrast-enhanced CT is crucial for managing hematomas [8], because it can pinpoint the source vessel of the bleeding, detect active bleeding, and determine the hematoma’s location. Extravasation of contrast medium enables the identification of ongoing bleeding or the vascular source, the location of hematoma wall enhancement, and adjacent anatomical structures at a glance, which helps in performing embolization or surgery [11,12]. Ultrasonography does not have complications, such as nephropathy, due to contrast medium and enables a straightforward identification of hematomas based on identifying echogenic particulate mobile contents or multiple internal septations. However, it has limitations in evaluating large hematomas or hematomas in unfavorable anatomic locations [10,13]. In this patient, ultrasonography could not visualize the huge mass in a single image. Furthermore, ultrasonography is not covered by health insurance for evaluating soft tissue masses in Korea. Therefore, we chose CT for the evaluation. In this report, enhanced CT revealed a subpectoral hematoma, no active bleeding, and a branch of the lateral thoracic artery located adjacent to the hematoma. These CT findings were instrumental in guiding surgical interventions.

Percutaneous transluminal angiography (PTA) is recognized for its safety and efficacy, as it enables simultaneous diagnosis and treatment. According to a study by Fior et al., PTA demonstrated an immediate success rate of 98.7% for embolization in patients with active bleeding [6]. However, the incidence of contrast-induced nephropathy was reported at 31%, and chronic kidney disease persisted in 2.5% of cases [6]. In this particular case, the patient was not successfully diagnosed or treated with PTA. The presence of a large hematoma necessitated surgical intervention for evacuation, as the risks associated with soft tissue necrosis from ischemic skin damage and heme toxicity were greater than the potential benefits of achieving hemostasis through compression.

The features of spontaneous chest wall hematoma reported in recent cases reveal that anticoagulation therapy was the cause in all cases. Most cases occurred in older adults with multiple comorbidities or in those with critical general conditions. Hemodynamic instability was resolved with appropriate interventions, and the patients were discharged. The source vessel of bleeding was often a main pedicle or a branch of the subclavian artery, axillar artery, thoracoacromial artery, or lateral thoracic artery adjacent to the chest wall. As presented in this report, some imaging studies did not identify the extravasating vessel (Table 2).

A possible reason why active bleeding or extravasation was not detected on CT angiography and PTA, yet was discovered during surgery, may be related to a microvascular origin, such as microangiopathy or small-vessel arteriosclerosis. Research indicates that a patient’s inflammatory state or comorbidities, such as aging, can lead to microangiopathy. This condition makes the microvasculature susceptible to injury from even minor traumas, such as coughing, rapid position changes, or the Valsalva maneuver, ultimately resulting in soft tissue hematoma [6,8]. In this report, the patient was encouraged to expectorate excess sputum, and it can be inferred that minor trauma or blood pressure spikes due to coughing may have caused momentary microvascular damage. This may be why extravasation of the main vessel was not detected on the imaging study.

Conservative treatment includes performing a compressive dressing in the expectation of hematoma resorption and correcting coagulopathy with volume resuscitation, transfusion, and supplementation with fresh frozen plasma. Although conservative treatment is effective, cases involving active bleeding, large hematoma volumes, or hemodynamic instability necessitate prompt identification of the cause of coagulopathy and rapid interventions to achieve an optimal outcome [14]. Common interventions include transcatheter arterial embolization and surgical drainage. In this regard, surgical exploration and drainage were critical interventions for large hematomas in this patient. It offers several benefits: it removes the source of inflammation, reduces the risk of infections such as abscess formation and bacteremia, and facilitates the identification and ligation of microvascular injuries that are not detectable on PTA.

The HAS-BLED risk score is used to predict the bleeding tendency in anticoagulated patients with atrial fibrillation [5]. It assesses various factors, including hypertension (systolic blood pressure > 160 mmHg), abnormal renal or hepatic function, history of stroke, bleeding history or predisposition, labile INR (time in therapeutic range < 60%), age over 75 years, use of drugs such as antiplatelets or nonsteroidal anti-inflammatory drugs, and alcohol consumption. A score of four points or higher signifies a high risk, with an annual bleeding risk of at least 4%. A score of 2 to 3 points indicates a moderate risk, with a bleeding risk of 2–4% per year, while a score of less than 2 points suggests a low risk, with a bleeding risk of 2% or less per year. The HAS-BLED score is typically used to predict bleeding risk in patients on anticoagulation therapy. However, Nopp et al. assessed the bleeding risk in ESRD patients using scores such as HAS-BLED, HEMORR2HAGES, and ATRIA [15]. The HAS-BLED score, while not perfect, showed the best result, with a C-statistic of 0.80 [15]. Based on this, we used the HAS-BLED score to predict the bleeding risk in this patient with ESRD but not on anticoagulation treatment. The patient discussed in this report had high blood pressure, abnormal renal function, a history of bleeding, and was older than 75 years, resulting in a score of 4 points, which indicates a high risk. Although the patient exhibited a bleeding tendency, no bruising was observed during the physical examination. The coagulation profile from the laboratory tests was within the normal range. Therefore, no further laboratory tests (e.g., coagulation factors) were performed, and the evaluation of the hematoma was delayed due to findings suggestive of an infection.

The patient described in this report exhibited a high-risk bleeding tendency, which was not attributed to common causes such as anticoagulant use. The primary suspected cause of the bleeding was uremic thrombocoagulopathy. This condition is known to lead to bleeding complications due to several factors: dysfunction in platelet aggregation, Von Willebrand factor abnormalities, reduced erythrocyte mass, and elevated levels of cyclic adenosine monophosphate or cyclic guanosine monophosphate [4,7,16]. Previous studies have revealed that blood urea nitrogen (BUN) levels above 35 mg/dL can be a predictor of spontaneous gastrointestinal bleeding, and BUN levels between 150 and 200 mg/dL are associated with spontaneous brain bleeding or an increased risk of bleeding episodes even with small trauma [16]. Although heparinization during hemodialysis has been reported to potentially induce spontaneous bleeding, the patient typically had a BUN level of 30 mg/dL, which rose to 50.3 mg/dL at the time the hematoma occurred, indicating uremic coagulopathy. It is important to note that not all dialysis patients experience spontaneous bleeding or are at risk of hematoma. However, it is hypothesized that spontaneous bleeding could be exacerbated by the presence of additional risk factors such as aging and hypertension, which contribute to a high HAS-BLED score.

In this case, unlike those in which anticoagulation therapy was the cause of most spontaneous soft tissue hematomas, no anticoagulant was used, and uremic coagulopathy was the main factor associated with a high bleeding risk. In this situation, a microvascular injury that can cause bleeding even with minor trauma or a blood pressure spike might have been the mechanism of spontaneous bleeding. In such cases, active bleeding may be missed due to the absence of extravasation on CT angiography or arteriography. However, if a large hematoma volume or hemodynamic instability is present, aggressive surgical exploration to evaluate the source vessel and drainage of a large hematoma can prevent complications such as skin ischemia or mass effect. This approach can serve as an effective treatment method to achieve outcomes.

## 4. Conclusions

Spontaneous chest wall hematomas are rare but critical complications, particularly in patients with end-stage renal disease undergoing hemodialysis. This case highlights the interplay of factors such as uremic coagulopathy, microvascular fragility, and high blood pressure, which contribute to an elevated risk of spontaneous bleeding.

For large hematomas or cases of hemodynamic instability, surgical evacuation is a crucial intervention. It ensures hemostasis by addressing microvascular injuries and prevents complications. In this case, the hematoma was successfully managed through a combination of surgical drainage and vessel ligation, without postoperative complications.

This report emphasizes the importance of the timely recognition of spontaneous soft tissue bleeding risk in ESRD patients undergoing hemodialysis. It also highlights the diagnostic and therapeutic approaches that can be utilized when bleeding occurs. Further research is warranted to optimize bleeding risk prediction tools and treatment protocols to improve outcomes in similar cases.

## Figures and Tables

**Table 1:** Laboratory test results.

Variable	Reference Range	Value
Hemoglobin (g/dL)	12–18	8.3
Platelet count (×10^3^/μL)	130–450	139
White blood cells (×10^3^/μL)	4.8–10.8	7.7
Neutrophil (%)	50–75	84.0
Lymphocyte (%)	20–40	9.0
Reticulocyte count (%)	0.5–2.0	4.80
Prothrombin time (s)	10–13.6	13.4
International normalized ratio	0.8–1.2	1.21
Activated partial-thromboplastin time (s)	22.5–34.5	31.0
Aspartate transaminase (U/L)	10–37	13
Alanine aminotransferase (U/L)	10–37	4
Blood urea nitrogen (mg/dL)	8–23	50.3
Creatinine (mg/dL)	0.5–1.3	3.04
Sodium (mEq/L)	136–146	129
Potassium (mEq/L)	3.5–5.1	4.5
Albumin (g/dL)	3.5–5.2	1.8
C-reactive protein (mg/dL)	0–0.3	8.04
Procalcitonin (ng/mL)	0–0.5	7.05

**Table 2:** Features of spontaneous chest wall hematoma cases.

Case (Author, Year)	Sex	Age	Comorbidities	Causes	Hematoma Location (Source Vessel)	Size (cm)	Treatment	Survival
Fuentes-Martín et al., 2022 [1]	Male	75	COVID-19	COVID-19 coagulopathy, enoxaparin	Left (subclavian-axillary artery branch)	14 × 13 × 7	Embolization	Yes
Fuentes-Martín et al., 2022 [1]	Female	96	COVID-19, hypertension, ESRD, arrhythmia	COVID-19 coagulopathy, edoxaban, enoxaparin	Left (cannot vascular identifying)	18 × 16 × 9	Embolization, surgical drainage	Yes
Yoon et al., 2021 [5]	Male	69	Acute coronary syndrome, hypertension, dyslipidemia	Ticagrelor, aspirin	Right (lateral thoracic artery)	(not mentioned)	Conservative treatment	Yes
Subedi et al., 2020 [2]	Male	79	Asthma, alcohol use disorder	Enoxaparin	Left (not mentioned)	17.5 × 15.9 × 6.5	Conservative treatment	Yes
Koklu et al., 2016 [9]	Female	79	Parkinson’s disease, hypertension, heart failure, acute kidney disease	Warfarin	Right (no mentioned)	14.7 × 14.2 × 8.6	Conservative treatment	Yes
Benbouchta et al., 2021 [8]	Female	72	Arrhythmia	Acenocoumarol	Left (cannot vascular identifying)	15.3 × 12.3	Surgical drainage	Yes

## Data Availability

No new data were created or analyzed in this study. Data sharing is not applicable to this article.
